# PKC translocation and ERK1/2 activation in compensated right ventricular hypertrophy secondary to chronic emphysema.

**DOI:** 10.1186/1472-6793-5-6

**Published:** 2005-05-05

**Authors:** Erick Avelar, Thunder Jalili, Li Dong, Joel Arvizo, Ping Hu, Sheldon E Litwin, John P Mattson

**Affiliations:** 1 Division of Cardiology, University of Utah, 30 N. 1900 E., Salt Lake City, UT 84132, USA; 2 Division of Nutrition, University of Utah, 250 S. 1850 E., #214, Salt Lake City, UT 84112, USA; 3 Department of Health & Exercise Science, Gustavus Adolphus College, 800 W. College Ave., St. Peter, MN 56082, USA

**Keywords:** signal transduction, echocardiography, pulmonary hypertension

## Abstract

**Background:**

Right ventricular hypertrophy (RVH) is an important complication of chronic lung disease. However, the signal transduction pathways involved as well as the physiological changes to the right ventricle have not been investigated. Emphysema was produced in male, Syrian Golden hamsters by intra-tracheal instillation of 250 IU/kg elastase (Emp, n = 17). Saline treated animals served as controls (Con, n = 15).

**Results:**

Nine months later, Emp hamsters had 75% greater lung volume, and evidence of RVH at the gross and myocyte level (RV:tibia length Emp 6.84 ± 1.18 vs. Con 5.14 ± 1.11 mg/mm; myocyte cross sectional area Emp 3737 vs. Con 2695 μm^2^), but not left ventricular hypertrophy. Serial echocardiographic analysis from baseline to nine months after induction of emphysema revealed increasing right ventricular internal dimension and decreased pulmonary artery acceleration time only in Emp hamsters. There was an increase in translocation of PKC βI and PKC ε from cytosolic to membranous cell fractions in RV of Emp hamsters. Phosphorylation of PKC ε was unchanged. Translocation of PKC α and βII were unchanged. Emp animals had a 22% increase in phospho-ERK 1/2, but no change in levels of total ERK 1/2 compared to Con.

**Conclusion:**

These data suggest that PKC βI, ε and ERK 1/2 may play a role in mediating compensated RVH secondary to emphysema and may have clinical relevance in the pathogenesis of RVH.

## Background

Emphysema affects nearly 3 million people and is responsible for nearly 16,000 deaths per year in the United States [1]. Pulmonary parenchymal disease may lead to increased pulmonary vascular resistance and elevated pulmonary arterial pressures. The development of pulmonary hypertension and RVH are markers of increased mortality in emphysema [2]. The problem of cor pulmonale is quite important since in the United States approximately 20% of hospital admissions for heart failure are caused by right heart failure. However, the precise changes in pulmonary hemodynamics, right ventricular structure and function in emphysema, as well as the underlying cell signaling pathways activated in this condition, remain unknown. We investigated these parameters using a hamster model in which emphysema is produced by intratracheal administration of elastase. This causes a reproducible and progressive form of bulbous emphysema characterized by increased lung volume and pulmonary parenchymal pathology similar to that of humans with emphysema [3].

Even though many studies have identified a critical role for many protein kinase C isoforms (PKC α, βI, βII, δ, ε) and extracellular regulated kinase (ERK) 1/2 in the pathogenesis of left ventricular hypertrophy, very few studies have examined these kinases in RVH. It has been previously reported that PKC α, βI, and δ activation is increased in RVH due to pulmonary artery constriction[4], and PKC α and δ activation increased during RVH induced by volume overload[5]. In contrast, there have been no studies to our knowledge regarding the role of ERK1/2 during RVH.

Given the numerous human and animal studies identifying PKC and ERK1/2 as critical mediators of left ventricular hypertrophy [6-12], and the aforementioned studies, we hypothesized that ERK1/2 and differential PKC isoforms would be activated in RVH secondary to emphysema. The progression of changes in right ventricular size and function over time using a noninvasive ultrasound approach, as well as pulmonary hemodynamics were also determined. We report here that hamsters developed moderate, well-compensated RVH characterized by an absence of fibrosis, normal contractility, and mildly increased pulmonary pressure after nine months with emphysema. Furthermore, hamsters with emphysema had marked translocation of PKC βI and PKC ε to membranous cell fractions, along with increased ERK 1/2 phosphorylation.

## Results

**Figure 1:**
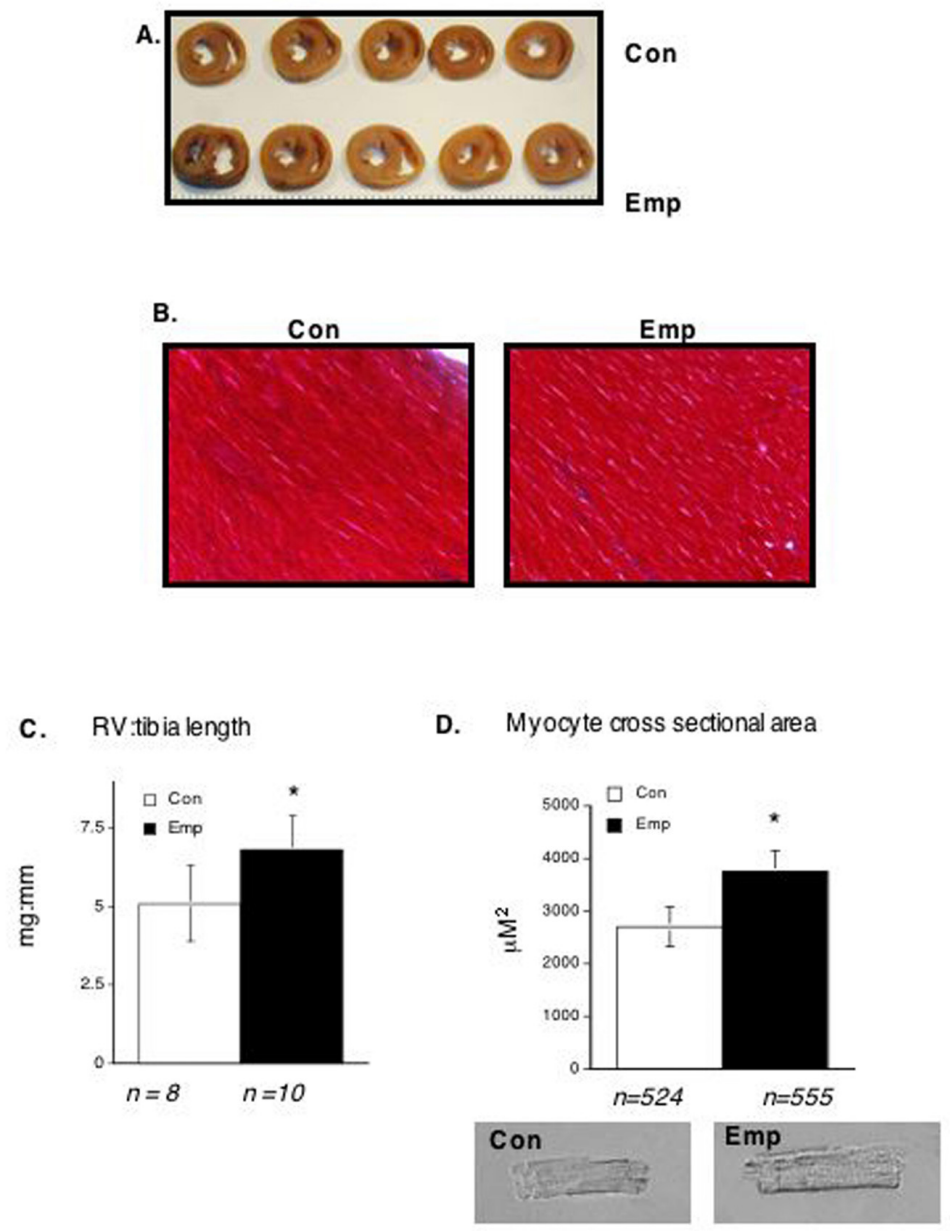
**A. **Gross cross sections of hearts from hamsters with emphysema and age matched controls. Hamsters with emphysema demonstrate a range of RV free wall thickness and chamber dilation, however all are larger than controls. Reference scale on bottom of picture is in mm. **B. **Histological sections of hearts were stained with Masson's Trichrome to visualize collagen content. No significant evidence of fibrosis were seen in Emp hamsters compared to Con. **C. **Right ventricle:tibia length is greater in Emp animals compared to Con. **D. **Myocytes cross sectional area was greater in right ventricles from Emp animals compared to Con. Data collected from individual myocytes (524 control, 555 Emp) collected from 7 Con and 5 Emp hamsters. Con, control; Emp, emphysema.

Total heart weights and RV weights were greater in Emp animals than Con, with the increased total heart weight in Emp being entirely due to the increase in RV mass (Table 1, Figure 1A &1C). Myocyte cross sectional area was greater in Emp right ventricles compared to controls (3737 μM^2 ^vs. 2695 μM^2^) (Figure 1D) as was myocyte perimeter length (Table 1). The presence of lung pathology and air trapping in the Emp animals was evidenced by a 75% increase in lung volume (Table 1).

**Figure 2:**
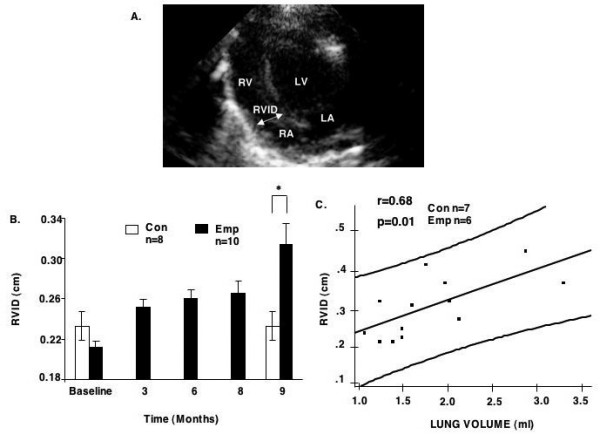
**A. **An example of the right ventricular internal diastolic dimension (RVID) as measured from the apical four-chamber view (RA, right atrium; RV, right ventricle; LA, left atrium; LV, left ventricle). **B. **RVID was measured at baseline, 3, 6, 8 and 9 months in the Emp group and at baseline and nine months in the Con group. Note the significant RV enlargement over time in the Emp group. RVID in the Emp group was significantly larger than the Con group after nine months (*p ≤ 0.05). **C. **There is a significant positive linear correlation between RVID and lung volume for both the Emp and Con groups after nine months. Con, control; Emp, emphysema.

Echocardiographic assessment documented progressive right ventricular enlargement over time in the Emp hamsters, with significantly greater right ventricular internal dimension (RVID) vs. Con after nine months of emphysema (Figure 2B). Histological examination of heart cross sections showed no differences in fibrosis in either right or left ventricles of Emp hamsters compared to Con (Figure 1B).

**Figure 3:**
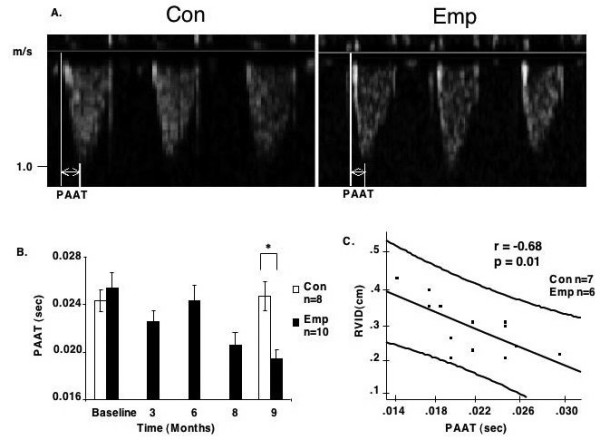
**A. **Representative examples of right ventricular outflow tract Doppler flow patterns in Con and Emp hamsters nine months after elastase or vehicle treatment. A more symmetric contour of the Doppler spectrum is seen in the Con group as compared to the Emp animals which show an earlier peak flow velocity. The points used for measurement of RVOT acceleration time are indicated. **B. **Right ventricular outflow tract Doppler spectra were measured at baseline, 3, 6, 8 and 9 months in the Emp group and at baseline and nine months in the Con group. Note the significant decrease in pulmonary artery acceleration time (PAAT) over time in the Emp group. Right ventricular outflow tract acceleration time in the Emp group was significantly shorter that the Con group after nine months (*p ≤ 0.05 vs. Con). **C. **Right ventricular internal diastolic dimension (RVID) vs. PAAT for both the Emp and Con groups after nine months. Note the significant inverse linear correlation between RVID and PAAT in the Emp hamsters. Con, control; Emp, emphysema.

There was a significant correlation (r = 0.68) between the RVID and lung volume measured (Figure 2C). Only trivial amounts of tricuspid valve regurgitation were found in any hamster at any time point and there was no detectable pulmonary insufficiency. Thus, RV and pulmonary artery pressures could not be estimated from the noninvasive studies. Right ventricular outflow tract (RVOT) Doppler profiles were adequate for analysis in all animals. RVOT acceleration time progressively shortened in the Emp animals and was significantly shorter compared to the Con after nine months (Figure 3A,B). There was an inverse correlation between the RVOTAT and RVID (r = - 0.68) at nine months (Figure 3C).

**Figure 4:**
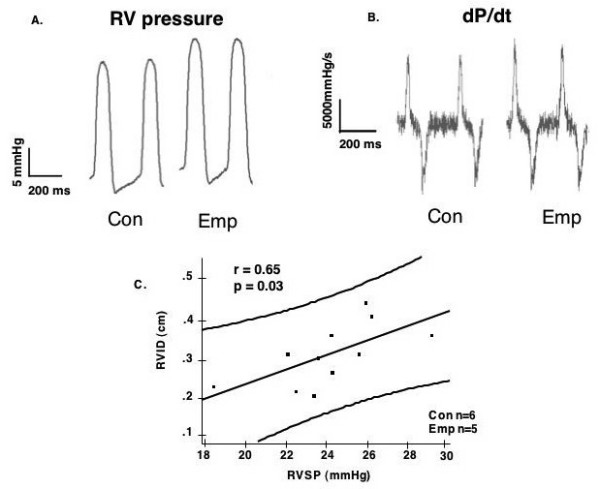
**A. **Representative examples of right ventricular pressure tracing in Con (n = 7) and Emp (n = 7) hamsters. Right ventricular systolic pressure (RVSP) was significantly higher in the Emp group. **B. **First derivative of RV pressure (dP/dt) obtained in Con (n = 7) and Emp (n = 7) hamsters. There was a higher +dP/dt in the Emp group vs. Con. **C. **There is a significant positive linear correlation between RVID and RVSP in Con and Emp groups suggesting that the increased RVSP, although mild, is enough to produce RV enlargement. Con, control; Emp, emphysema.

There was a mild, but statistically significant increase in right ventricular systolic pressure (RVSP) after nine months of emphysema (Figure 4A, Table 1). There was a strong positive correlation (r = 0.65) between RVSP and RVID (Figure 4C). Emp hamsters also had a slightly greater +dP/dt and similar -dP/dt compared to the Con group, implying a mostly normal pattern of RV contraction & relaxation (Figure 4B, Table 1).

**Figure 5:**
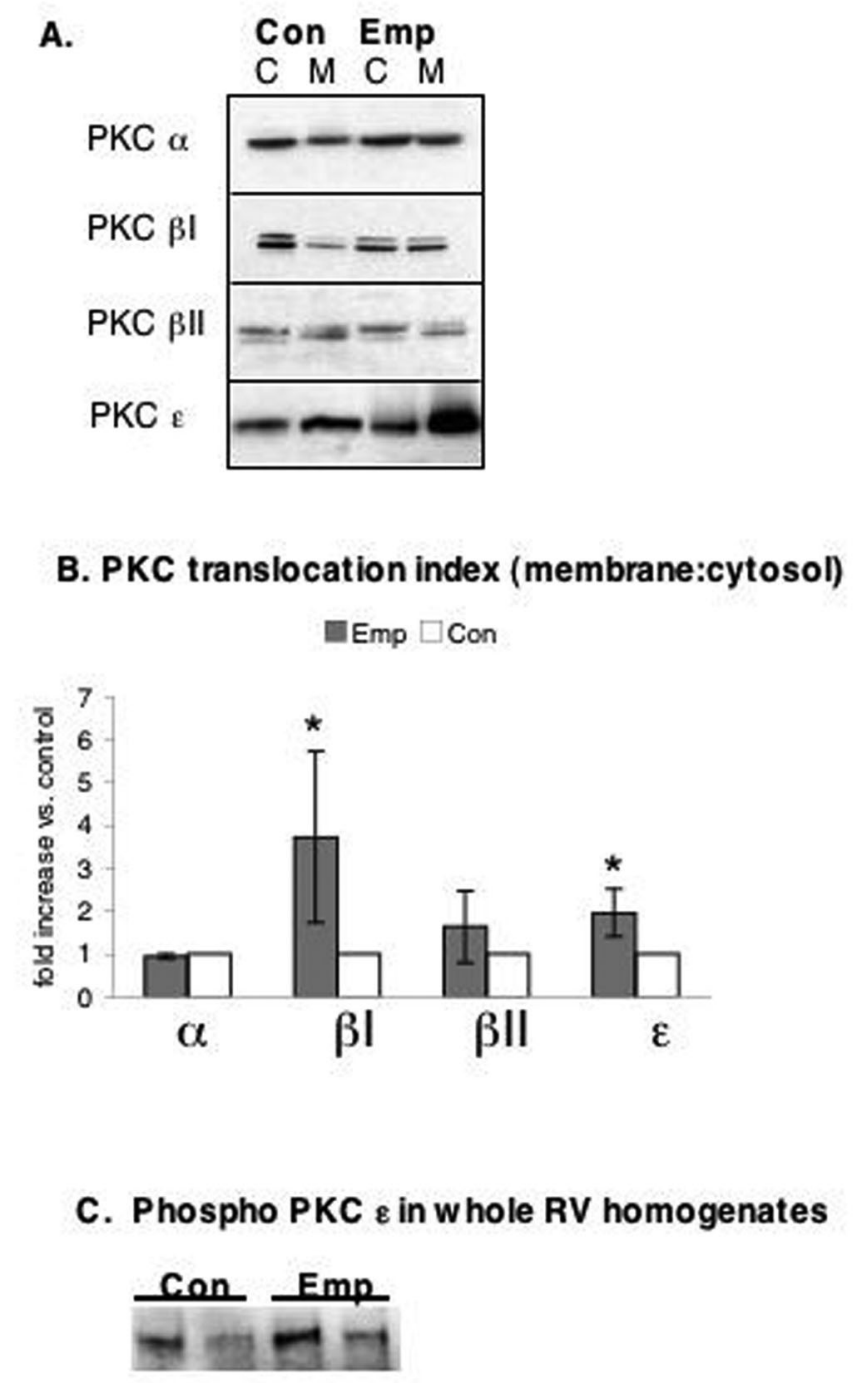
**A. **Protein kinase C (PKC) immunoreactivity during right ventricular hypertrophy in hamsters with emphysema. Membranous (Mem) and cytosolic (Cyt) fractions were prepared from hamster right ventricle. Representative immunoblots for PKC ε and PKC βI demonstrate increased translocation in Emp, while PKC α and βII were unchanged. Translocation was quantified by taking ratio of membrane to cytosolic levels (M:C) of each isoform. Average PKC ε translocation index (of three separate trials) was 1.14 ± 0.11 for Con (n = 5) and 2.22 ± 0.33 for Emp (n = 5) animals. **B**. Graph of M:C illustrates fold change normalized to controls. Fold changes are averages of three separate experiments for each PKC isoform. **C. **Levels of total phosphorylated PKC ε from whole right ventricular homogenates were unchanged among Con and Emp hamsters. *p ≤ 0.05. Con, control; Emp, emphysema.

No differences were detected in translocation of PKC isoforms α and βII between Emp and Con hamsters, as evidenced by the unchanged membrane-to-cytosol ratio (Figure 5A,B). There was a shift in distribution of PKC βI to membrane fractions resulting in an increase in translocation (Figure 5A,B). There was also a 95% increase in translocation of PKC ε in the RV of Emp hamsters (Figure 5A,B). The increase in PKC ε translocation was characterized by unchanged cytosolic fractions in coordination with a 110% increase in PKC ε found in the membrane fraction (Figure 5A,B). The marked increase in membrane levels of PKC ε indicates that protein expression of ε was also increased, with a greater proportion of that isoform being distributed in the membrane fractions. The average translocation index (membrane:cytosol) was 1.14 ± 0.11 for Con and 2.22 ± 0.33 for Emp animals. Levels of phosphorylated PKC ε from whole RV homogenates were quite variable, but means were similar between Con and Emp animals (Figure 5C). Increased levels of PKC ε membrane translocation, however, suggest that more of the phosphorylated PKC ε may be in the membrane fraction of Emp hamsters rather than the cytosol. PKC βI phosphorylation was not determined in this study since antibodies specific for phosphorylated PKC βI are not commercially available.

**Figure 6:**
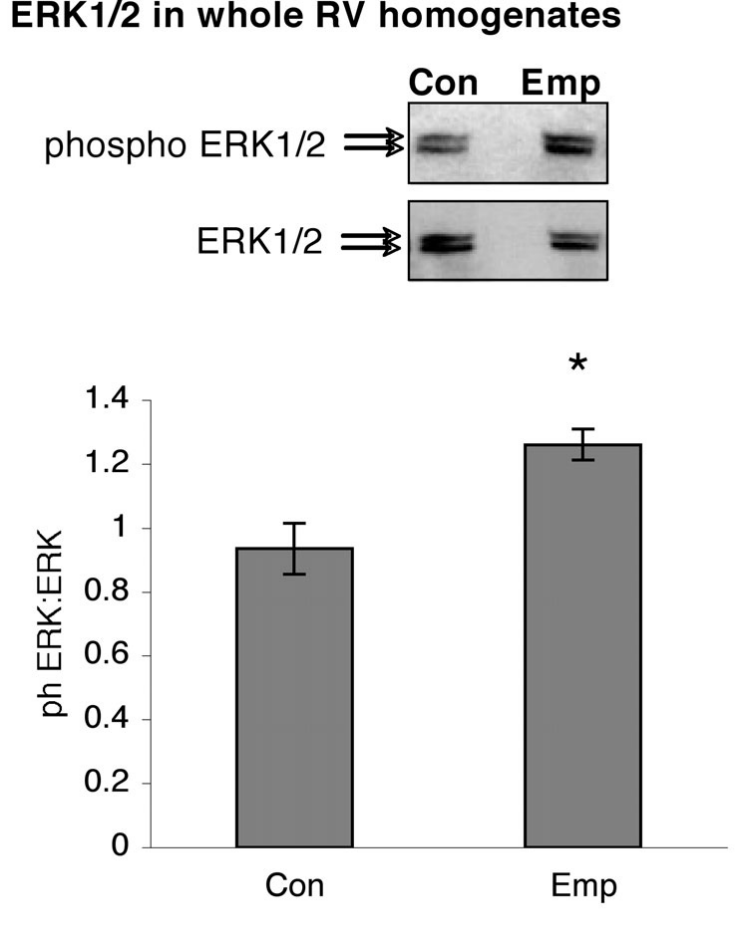
Immunoblots of whole RV homogenates demonstrating a 34% increase in phERK1/2 to ERK1/2 ratio. Con, control; Emp, emphysema.

RV homogenates from Emp hamsters had increased phospho-ERK1/2 levels, resulting in a 34% increase in the ratio of phospho ERK1/2 to ERK1/2 (Figure 6). Total JNK levels were similar between Con and Emp groups (data not shown). Phospho-JNK was not detected in RV homogenates from either Con or Emp hamsters. This result most likely reflects insensitivity of the antibody used against hamster phospho-JNK and should not be taken as evidence that JNK is unimportant in the development of RVH.

## Discussion

Despite the morbidity and mortality, associated with right heart failure, the pathogenesis of right ventricular hypertrophy has been studied much less than left ventricular disease. Evidence from clinical observations and animal studies suggest that the right ventricle does not respond to stress in precisely the same fashion as the left ventricle. Most patients with L-transposition of the great arteries, a condition in which the anatomic right ventricle serves as the systemic ventricle from birth, eventually develop severe RV failure. This usually occurs before the age of 30 [13]. Thus, clinical evidence suggests that the genetic program of the right ventricle is not designed to face an increased work load even when the adaptation is very gradual. Several animal studies also show different responses of the right and left ventricles to pressure overload [14,15].

The most commonly studied small animal model of right heart failure is the monocrotaline treated rat. Monocrotaline injection produces severe pulmonary hypertension over a period of several weeks [16]. The elastase treated hamster may be more closely related to clinical features of RVH secondary to emphysema than other models using pulmonary hypertension/overload for two reasons. First, the lung injury develops much more slowly with different degrees of hypoxia leading to varying levels of pulmonary artery constriction. This is substantiated by only modest echocardiographic changes (increased RVID and shortened RVOTAT) found in this study in the first six months after elastase treatment (Figure 2B, 3B). Second, the degree of pulmonary hypertension is less severe than that which is usually seen after monocrotaline injection where pulmonary pressure often exceeds 65 mmHg within six weeks [16,17]. Clinically, most patients with chronic obstructive pulmonary disease such as emphysema have mild or moderate pulmonary hypertension at rest [18], perhaps more similar to the mild increases in RV systolic pressure found in Emp animals in the present study. There have been several hypotheses to explain the development of pulmonary hypertension in the setting of emphysema, with hypoxic vasoconstriction being the most supported mechanism [19]. Other factors such as *in situ *thrombosis and polycythemia may play a role as well [18].

In the present study RV from the EMP animals had slightly increased +dP/dt, normal -dP/dt, and lack of fibrosis in histological sections, together indicating presence of compensated RVH. Despite these changes, we were unable to detect anything more than trivial degrees of tricuspid regurgitation in the Emp hamsters. Interestingly, even in the absence of tricuspid regurgitation, the RVOT acceleration time showed an inverse correlation with RV size. Based on these data it seems likely RVOT Doppler spectra may be a useful surrogate marker for determining the presence of early or mild forms of pulmonary hypertension in the absence of tricuspic valve regurgitation.

This is the first study to demonstrate PKC βI, PKC ε and ERK1/2 activation in RVH secondary to emphysema. In total we evaluated PKC α, βI, βII, δ, and ε in this study as these isoforms have all been implicated in the pathogenesis of cardiac hypertrophy and ventricular dysfunction in cell culture, insult driven, and transgenic animal models[6,7,12,20]. The unchanged levels of cytosolic PKC ε coupled with a marked increase in membrane PCK ε indicate that expression levels were upregulated in Emp. Thus, it appears right ventricular PKC ε response to emphysema is more complex than a simple re-distribution of basal levels, and involves greater production of PKC ε protein. With specific regard to PKC ε, several lines of evidence support a role for PKC ε in the development of cardiac hypertrophy as reviewed by Sabri et al. [21]. Recent studies using transgenic mice with cardiac specific overexpression of PKC ε now suggest that low to moderate PKC ε activation, characterized by a 6 fold increase in translocation [7] or 228% increase in PKC activity [22] result in physiological cardiac hypertrophy with normal cardiac function. In contrast, a high degree of PKC ε activation, characterized by a 34 fold increase in translocation [7] or 452% increase in activity [22] result in pathological hypertrophy characterized by poor contractility and heart failure. Furthermore, it has also been shown that moderate activation of PKC ε in vivo by it's RACK (Receptor for Activated C Kinase) increases translocation of PKC ε by only 20% and results in mild compensated hypertrophy [23]. It is thought that in the case of excessive PKC ε activation, heart failure is mediated by PKC ε induced cross activation of PKC βII, which has been previously demonstrated to cause a heart failure phenotype in transgenic mice [9,12]. The present study found a 95% increase in PKC ε translocation, but no increase in PKC βII in the RV of Emp hamsters. This appears to fall in to the range of "moderate" levels of PKC ε translocation that have been associated with compensated/physiological hypertrophy in the aforementioned studies. In contrast with our results, another study using rats subjected to pulmonary artery constriction for 3 weeks found greater membrane levels of PKC α and δ, but no change in PKCε [4]. It is possible that greater RV systolic pressure after pulmonary constriction (44 mmHg in pulmonary constricted rats vs. 26 mmHg in Emp hamsters) resulted in differential activation of PKC isoforms. Other differences that may have contributed to these contrasting results include the duration of the study (3 weeks vs. 9 months), the degree of RVH developed (much less in Emp hamsters), and the species studied.

An analysis of total PKC ε phosphorylation in whole RV homogenates found similar levels in both Emp and Con hamsters, however, these samples also demonstrated significant variation in phosphorylation state among individual hamsters. This may be due to the phospho specific PKC ε antibody used, which was derived against a human epitope, and may have had reduced specificity against the hamster protein. We used an antibody that recognized PKC ε phosphorylated at Ser 729, an amino acid in the C-terminal hydrophobic motif. Ser 729 is autophosphorylated by PKC ε itself following PDK-1 dependent phosphorylation of Thr 566[24]. PKC ε phosphorylated at this site is recognized as mature and catalytically active[25].

Previous studies using neonatal myocytes, animals models, and transgenic mice indicate that PKC β isoforms, particularly βII, also are key mediators in the pathophysiology of cardiac hypertrophy and heart failure [12,26]. While there are no transgenic models that specifically implicate PKC βI in pathogenesis of hypertrophy, other studies have found activation to be present during pressure overload hypertrophy in vivo [27,28]. Translocation of PKC βI may also be clinically relevant since increased activity in PKC β (I and II) has been reported by Bowling et al. in failing human hearts [10]. Our data is partially consistent with these finding as we found a significant increase in translocation of PKC βI, but no changes in PKC βII in hypertrophied RV of Emp hamsters. Since previous studies using transgenic mice indicate that PKC βII appears to produce a heart failure phenotype [9,12], we speculate that the lack of PKC βII translocation in this study may due to the compensated nature of the hypertrophied right ventricle at the time of analysis. Taken together, our data on PKC translocation suggest that future studies using pharmacological agents that can selectively inhibit PKC isoforms will help confirm a specific role for PKC in this form of RVH, and give insight into possible therapeutic interventions to limit the progression of RVH secondary to emphysema.

Levels of phospho ERK1/2 were 22% greater, along with a 34% increase in phospho ERK:total ERK in Emp compared to Con. These are mild increases compared to other in vivo studies using pressure overload imposed upon the left ventricle [29-31]. The importance of ERK signaling has been established in the hypertrophic response with recent evidence supporting a role for adaptive hypertrophy and survival as opposed to heart failure [32,33]. Given that previous studies have reported more vigorous ERK activation in response to pressure overload, it is difficult to determine the true physiological relevance of our ERK data with regards to the pathogenesis of right ventricular hypertrophy. However, it is possible that the degree of ERK activation may fluctuate over time as hypertrophy develops and progresses. However, a potential limitation in our phospho PKC ε and ERK1/2 analyses is that whole tissue homogenates were used for experiments. Thus we cannot assess the contribution from the non muscle fraction where activation of EGF receptor and receptor tyrosine kinases may be operative. Another limitation is the analysis of signal kinases after the establishment of RVH. While these data correlate PKC βI and ε translocation, and EKR1/2 activation with RVH, they do not confirm these signal kinases in mediating development RVH.

## Conclusion

This study underscores the integrated nature of the cardio-pulmonary system such that experimentally induced lung disease significantly impacted cardiac physiology. To the best of our knowledge, the present study is the first to have identified activation of PKC and ERK/12 in RVH secondary to emphysema, and to describe the progressive nature of alterations in the right ventricle during progression of emphysema. Taken in context of previous studies, these data support the hypothesis that PKC and ERK may play an important role in the pathophysiology of RVH secondary to emphysema. It is also evident that after nine months of emphysema in this model, the RV is in a compensated pattern of hypertrophy with preserved systolic function. Overall, we believe that our data regarding right ventricular structure and function in this model of chronic parenchymal lung disease with mild RV pressure overload complement data from other studies of acute severe pulmonary hypertension induced with monocrotaline. Additional studies determining status of PKC and ERK immediately prior to the development of RVH in this model would help further define their relevance as critical mediators of RVH secondary to emphysema. Data from the present study, as well as future studies, may be useful to identify new diagnostic and treatment paradigms for right ventricular overload.

## Methods

### Emphysema model

Male Syrian Golden hamsters (9 week old, 100–121 g body weight) were maintained on a 12:12-h light-dark cycle, and supplied with rodent chow and water ad libitum. After one-week habituation period, the animals were divided into control (Con, n = 15) and emphysema (Emp, n = 17) groups at random. Under deep anesthesia with ketamine/xylazine (150/7.5 mg/kg im), either saline (0.3 ml/100 g body wt) or porcine elastase [25 IU/100 g body wt (Sigma Chemical, St. Louis, MO) in 0.3 ml of normal saline] was instilled intratracheally using a 27-gauge hypodermic needle as previously described [34].

### Echocardiographic measurements

Parameters of cardiac structure and function were determined with transthoracic echocardiography (n = 8 con and n = 10 Emp hamsters) as previously described [35,36]. Hamsters were anesthetized using chloral hydrate (300 mg/kg, intramuscularly) and the chest was shaved. Two-dimensional and Doppler imaging were performed using a 4.5 – 10 MHz transducer (Vivid Five, General Electric). Right ventricular internal diastolic dimension (RVID) was measured in the apical four chamber view just below the level of the tricuspid valve. Pulsed-wave Doppler of the pulmonary outflow tract was recorded in the parasternal short axis view at the level of the aortic valve. The sample volume was placed proximal (3 mm) to the pulmonary valve leaflets and aligned to maximize laminar flow and the right ventricular outflow tract acceleration time (RVOTAT), velocity-time integral and ejection time were measured. Acceleration time was measured from the onset of systolic flow to highest laminar velocity in the spectral signal (Figure 2A). The tricuspid valve was examined for the presence of tricuspid regurgitation (TR) with color and continuous-wave Doppler in the parasternal short axis and apical four-chamber views. Digital images were analyzed off-line (EchoPAC, General Electric). An echocardiographer who was blinded to the treatment group performed and analyzed all studies. All measurements represent the means of three cardiac cycles.

### Right heart catheterization

Following the final echo (nine months after elastase treatment) while still under anesthesia with chloral hydrate the right jugular vein was exposed and a temperature-calibrated 1.4 F micromanometer-tipped catheter (Millar Instruments) was inserted into the vein and advanced in retrograde fashion into the right ventricle. Heart rate, right ventricular pressure and first derivative of pressure (dP/dt) were recorded.

### Tissue harvesting

Nine months following elastase injection, hamsters were euthanized and the hearts and lungs were removed. Whole hearts were quickly weighed and then placed in 4°C saline where the RV was dissected and weighed separately. In a subgroup of animals (Con, n = 8; Emp n = 10) the RVs were immediately frozen in liquid N_2 _and stored separately at -80°C until further analysis. A saline displacement technique was used to measure excised lung volume at 0-cm H_2_O airway pressure as previously described [37]. In addition, for each hamster, the left tibia was dissected and the length was measured so that RV mass and total heart mass could be normalized to the tibial length as has been previously described and validated[38].

### Histological Assessment and myocyte measurements

In another subgroup of hamsters (Con, n = 7; Emp n = 5), the hearts were rinsed in ice cold saline and then fixed by immersion in formalin. Short axis slices from the mid wall level of hearts were cut in 10 μM sections from paraffin embedded tissue. Masson's trichrome-stained sections were examined by a pathologist (blinded to the animal identifications) and evaluated for presence of fibrosis.

Sections of RV adjacent to the portion used for histology were subjected to a previously reported technique designed to dissociate myocytes from fixed tissues [39]. Briefly, small pieces of the formalin-fixed RV tissue (~2 × 2 mm) were minced and incubated for 2 h in a 50% KOH solution (w/v made in 100 mM PBS) at 37°C. The partially dissociated tissue was then placed in a 2% trypsin solution for 3 hours and mechanically teased apart to produce mostly single myocytes. Samples were then centrifuged at1000 RPM for 5 min and the pellet containing intact myocytes was recovered. Myocytes were placed on glass slides and multiple fields from each heart were photographed with a digital camera at 20×. Right ventricular cell surface area and perimeter length was measured using NIH image software (V. 1.63) from 555 control and 524 Emp myocytes.

### Preparation of RV homogenates for PKC immunoblotting

Cardiac lysates containing cytosolic and membrane proteins were prepared from the hamster RV frozen at -80°C as previously detailed [6]. All extraction procedures were performed at 4°C. Protein concentration of cell fractions were determined using a Bio Rad Protein assay (Bio-Rad, Hercules, CA) with bovine serum albumin (BSA) as a standard.

### Preparation of RV homogenates for ERK 1/2, JNK, phospho PKC ε, and phospho c-Raf immunoblotting

The RV was homogenized with a tissuemizer in 1 ml of ice-cold RIPA buffer [50 mM Tris-HCl (pH 7.4), 150 mM NaCl, 1% Nonidet P-40, 0.25% sodium deoxycholate, 1 mM sodium orthovanadate, 1 mM NaF, and 10 μl/mL Sigma protease inhibitor cocktail (Sigma, St. Louis, MO, cat. #P-8340)]. Samples were then sonicated twice on ice and centrifuged at 11,000 × g for 10 mins at 4°C. Supernatants were recovered and stored at -80°C for subsequent immunoblotting. Protein concentration of RV lysate was determined using a Bio Rad Protein assay (Bio-Rad, Hercules, CA) with bovine serum albumin (BSA) as a standard.

### Western blotting, transfer and densitometry

Electrophoresis and transfer of proteins to PVDF membranes were done as previously described [6,29]. Gels were loaded with 10 μg per lane for PKC α, βII, and ε experiments, 20 μg per lane for PKC βII experiments, and 50 μg per lane for ERK and JNK experiments. All PKC antibodies were purchased from Santa Cruz Biotechnology (Santa Cruz, CA) all other antibodies / reagents were purchased from Cell Signal Technology (Beverly, MA). Primary antibody directed against PKC α, βI, βII, and ε were incubated overnight at 4°C in a 1:1000 dilution. Antibodies directed against total and phosphorylated ERK 1/2 & JNK, phospho c-Raf, and phospho PKC ε (recognizing phosphorylated Ser 729) were incubated at a 1:1000 dilution for 48 h at 4°C in 5% BSA Tris buffer with 0.05% Tween-20. Secondary antibody conjugated to horseradish peroxidase was incubated for 1 h at 1:10,000 dilution. Signals were visualized by enhanced chemiluminescence. Relative band density on film was measured with a scanner using NIH 1.63 image software (National Institutes of Health, Rockville, MD).

### Statistical analysis

For all data except echocardiographic data, an unpaired Student t-test was performed to detect the difference between Emp and Con animals using SPSS V. 10 for Macintosh (Chicago, IL). For changes in echocardiographic parameters over time, a general linear model with repeated measures was used. Pearson's correlation coefficient was used to describe the relationship among the echocardiographic variables, hemodynamic data, and gross anatomical measurements. Significance was accepted at p ≤ 0.05 for all tests. All values are expressed as mean ± SD.

## Authors' contributions

TJ helped supervise the scientific direction of project, assisted with immunoblots, conducted histology studies, analyzed immublots, histology, myocyte, animal morphometrical data, and drafted the manuscript. EA conducted serial echocardiographs and analysis, and drafted the manuscript. LD did the immunoblots and assisted in data analysis. JA determined myocyte dimensions. SEL helped supervise the scientific direction of the project, assisted with the echocardiographs and analysis, and helped draft the manuscript. JPM conceived of the study, prepared the hamster model, assisted in echocardiographs, and helped draft the manuscript.

## Figures and Tables

**Table 1:** Organ weights, right ventricle analysis, and heart rates of hamsters with emphysema.

	**Control**	**n**	**Emphysema**	**n**
Initial Body Wt. (g)	113 ± 9	15	112 ± 6	17
Final Body Wt. (g)	133 ± 16	15	132 ± 20	17
Lung Volume (mL)	1.43 ± 0.20	15	2.51 ± 0.68*	17
Heart Wt. (mg)	544 ± 78	15	616 ± 90*	17
Heart:tibia length (mg:mm)	19.4 ± 2.6	15	21.2 ± 3.2	17
RV Wt. (mg)	145 ± 33	8	197 ± 34*	10
Heart Rate (beats/min)	192 ± 30	7	190 ± 27	7
RVSP	22 ± 2	7	26 ± 2*	7
RVEDP	0.4 ± 0.4	7	0.9 ± 0.9	7
+dP/dT	1016 ± 162	7	1214 ± 139*	7
-dP/dT	-827 ± 142	7	-944 ± 114	7
Myocyte perimeter length (μM)	250 ± 11	5	302 ± 18*	5

*p ≤ 0.05 *RV*, right ventricle; *RVSP*, right ventricular systolic pressure (mmHg); *RVEDP*, right ventricular end-diastolic pressure (mmHg); *dP/dt*, first derivative of pressure (mmHg/sec).
